# Supplementary material for: Multi-scale turbulence simulation suggesting improvement of electron heated plasma confinement
Source: Nat Commun. 2022 Jun 7;13:3166. doi: 10.1038/s41467-022-30852-0 (PMC9174228; doi:10.1038/s41467-022-30852-0)
Supplement: Supplementary file 1 — Supplementary Information [file 41467_2022_30852_MOESM1_ESM.pdf]

Supplementary information

Multi-scale turbulence simulation suggesting improvement of electron heated plasma confinement

Shinya Maeyama*, Tomo-Hiko Watanabe, Motoki Nakata, Masanori Nunami, Yuuichi Asahi & Akihiro Ishizawa

*smaeyama@p.phys.nagoya-u.ac.jp

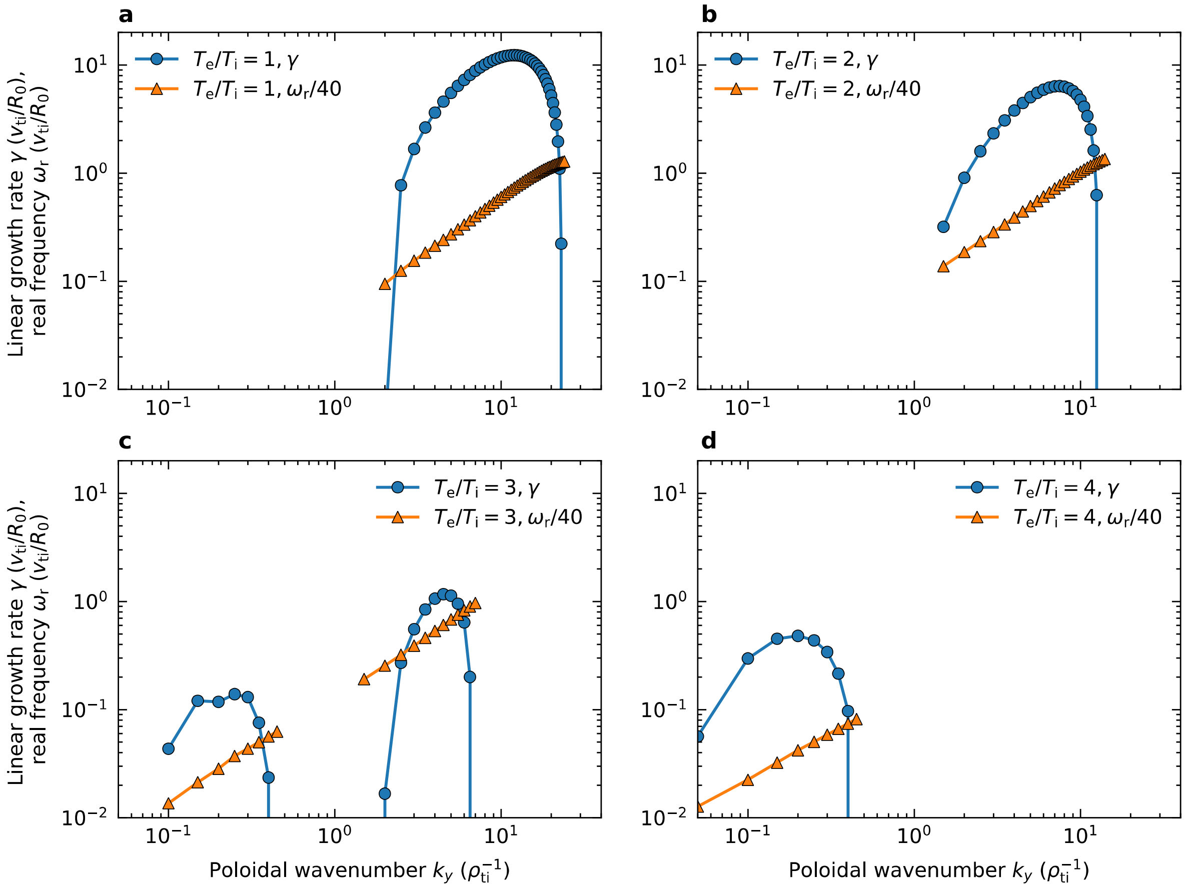

Supplementary Fig. 1 | Linear dispersion relation for different temperature ratio T_e/T_i. Linear growth rates γ (blue) and real frequencies ω_r (orange) are plotted as functions of the poloidal wavenumber k_y (for $k_x = 0$). Low-wavenumber modes at $k_y < \rho_{ti}^{-1}$ are trapped electron modes (TEMs), whereas high-wavenumber modes at $k_y > \rho_{ti}^{-1}$ are electron temperature gradient (ETG) modes. As the temperature ratio T_e/T_i increases (from **a** to **d**), the ETG modes are stabilised, whereas the TEMs are destabilised.

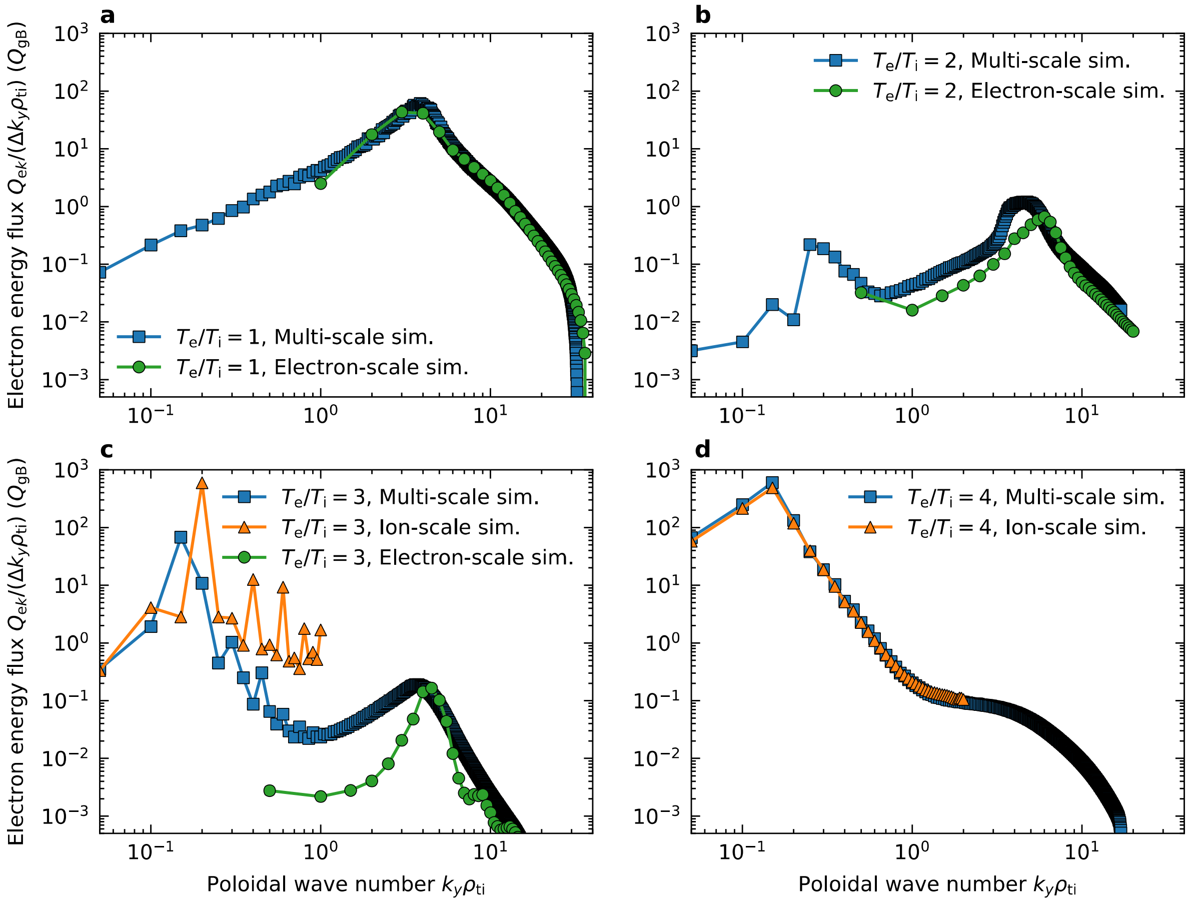

Supplementary Fig. 2 | Poloidal wavenumber spectra of the time-averaged electron energy flux Q_e for different temperature ratio T_e/T_i. The multi-scale TEM/ETG turbulence simulation result is plotted using a blue line. Results of a single ion-scale TEM turbulence simulation ($k_y\rho_{ti} \leq 1$) and single electron-scale ETG turbulence simulation ($k_y\rho_{ti} \geq 0.5$) are plotted using orange and green lines, respectively. **a**, For $T_e/T_i = 1$ case, ETG turbulence is well resolved in both of multi-scale and electron-scale simulations. **b**, For $T_e/T_i = 2$ case, excitation of linearly stable TEMs around $k_y\rho_{ti} \sim 0.3$ is observed, whereas ETG turbulence at $k_y\rho_{ti} \geq 1$ still dominate turbulent transport. **c**, $T_e/T_i = 3$ case is thoroughly reported in the main manuscript. **d**, For $T_e/T_i = 4$ case, TEM turbulence is well resolved in both of multi-scale and ion-scale simulations. There seems to be a small bump of the spectrum in high-wavenumber range $k_y\rho_{ti} \sim 4$, corresponding linearly stable ETG modes.
